# Are headaches different from other chronic pains?

**DOI:** 10.1007/s10194-011-0406-5

**Published:** 2011-12-21

**Authors:** Joanna M. Zakrzewska

**Affiliations:** Eastman Dental Hospital, UCLH NHS Foundation Trust, London, UK

Dear Editor,

Reading the editorial “Time to act on headache disorders” [1] begs me as an oral physician [(medical and dentally trained, running an orofacial pain service, member of International Association for the Study of Pain (IASP) and International Headache Society (IHS)] to ask a few questions.

Why are headaches a separate entity from other chronic pain? What are the boundaries for the head? How do professionals look at them? How do patients interpret them? The oral cavity and structures of the lower part of the face remain to a large extent the domain of the dental profession. Medical students rarely have training in this field [2]. One needs only to compare the criteria for diagnosis of temporomandibular disorders (TMD) in the classifications of IASP and IHS to those of the TMD Research Diagnostic Criteria, to see vast differences [3]. Where do these disorders fit?

The speciality of pain medicine is relatively young, IASP was founded in 1973 and IHS in 1981 with very similar aims but to include conditions of the head and neck only. The former is truly multidisciplinary IHS is nearly exclusively the domain of neurologists.

The co-morbidities such as depression and anxiety are the same for all chronic pain patients. TMD pain has been shown through case control studies to be linked with migraine, back pain, fibromyalgia and is an example of central sensitisation [4]. Pain management, therefore, needs input from a wide variety of specialties including anaesthesia, neurology, rheumatology, neurosurgery, orthopaedics, dentistry through to liaison psychiatry, physical therapy, psychology, occupational therapy. This need for multidisciplinary team (MDT) approach has resulted in pain management programs which have been shown through systematic reviews to be valuable. Yet in the headache field Gaul et al. [5] state that acceptance for an MDT approach in headache treatment is only now “gaining acceptance”.

IASP have declared this year the global year of headache in order to increase awareness of these conditions and the two organisations have worked together to produce a series of fact sheets for global use http://www.iasp-pain.org/Content/NavigationMenu/GlobalYearAgainstPain/GlobalYearAgainstHeadache. This would seem an excellent time to work towards unification to improve the global burden of pain.
